# Vasculopathy-related cutaneous lesions and intrahepatic cholestasis as synchronous manifestations in a COVID-19 patient; a case report 

**Published:** 2020

**Authors:** Amir Sadeghi, Arash Dooghaie Moghadam, Pegah Eslami, Ali Pirsalehi, Sina Salari, Elham Roshandel

**Affiliations:** 1 *Gastroenterology and Liver Diseases Research Center, Research Institute for Gastroenterology and Liver Diseases, Shahid Beheshti University of Medical Sciences, Tehran, Iran *; 2 *Assistant Professor of Medical Oncology, Hematology and Bone Marrow Transplantation; Taleghani Hospital, Shahid Beheshti University of Medical Sciences, Tehran, Iran*; 3 *Hematopoietic Stem Cell Research Center, Shahid Beheshti University of Medical Sciences, Tehran, Iran *

**Keywords:** COVID-19, cholestasis, liver, vasculitis, cutaneous lesion, skin

## Abstract

Today, COVID-19 pneumonia causes global concern. The World Health Organization (WHO) has reported many mortalities from this disease all around the world. Therefore, recognizing new cases of COVID-19 is crucial during this pandemic. Many studies have shown that COVID-19 has a broad spectrum of signs and symptoms, including GI and cutaneous manifestations. Previous studies have reported liver enzyme changes as well as diarrhea as a common GI manifestation of COVID-19. However, there are few reports about COVID-19 synchronous cutaneous and liver involvement. Additionally, there are few reports about intrahepatic cholestasis in COVID-19 patients. In this article, a confirmed case of COVID-19 with vasculopathy-related cutaneous manifestation and liver cholestasis is reported. A 67-year-old Iranian woman was admitted to Taleghani Hospital with epigastric pain, vomiting, anosmia, rising liver enzyme levels, fever, itching, and skin rashes. Skin and liver biopsies were performed during the patient’s admission; the results suggested vasculopathy-related cutaneous lesion and liver cholestasis. Plasmapheresis was initiated and all manifestations disappeared after treatment. All atypical presentations, including cutaneous lesions and liver manifestations, should be considered as COVID-19 and evaluated.

## Introduction

 Currently, the COVID-19 pandemic is recognized as a major cause of global concern. As of July 20, 2020, the 182th coronavirus disease (COVID-19) situation report published by the WHO reported about 14,350,000 accumulated cases and approximately 600,000 mortalities globally (1). To date, several manifestations of COVID-19 have been explained. Generally, COVID-19 is known as a viral pneumonia accompanied by fever, dry cough, and chest CT-scan changes (2). Some studies have also reported anosmia and neurological and cardiovascular manifestations with COVID-19 (3). 

For the first time, Guan et al. reported that COVID-19 might have cutaneous manifestations; however, they did not describe the characteristics of the cutaneous lesions (4). Several studies later reported skin involvement and vasculopathy-related cutaneous manifestations in COVID-19 patients (5). From the onset of COVID-19 in Wuhan, GI manifestations including liver injury and diarrhea have been important presentations (6-8). Nevertheless, no report has reported liver cholestasis in COVID-19 patients (6, 8, 9).

Currently, there are few reported cases of synchronous cutaneous and liver involvement and no previous report about liver cholestasis in COVID-19. Recognizing all COVID-19 features is important to controlling the pandemic and treating infected patients. Thus, we report herein a confirmed case of COVID-19 with vasculopathy-related cutaneous manifestation and liver cholestasis. 

## Case Report

**Figure 1:**
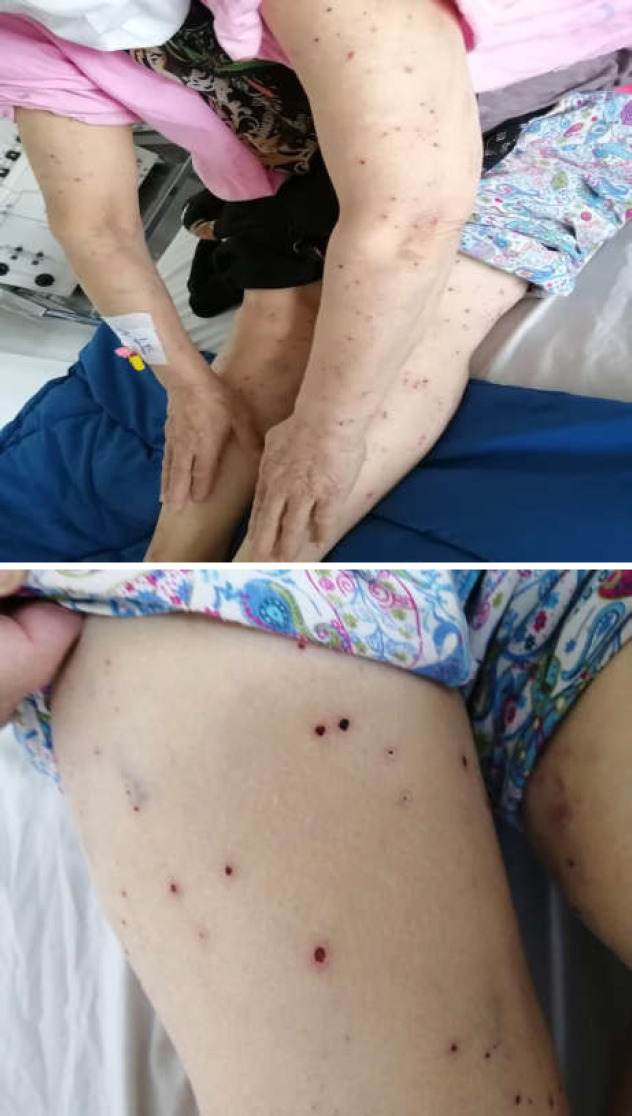
Painful red nodular lesions, three days after admission on upper and lower limbs with diagnosis of vasculitis

A 67-year-old Iranian woman presented to the emergency ward of Taleghani Hospital, a tertiary referral hospital for COVID-19 in Tehran, Iran. About 2 weeks before referring to our center, she experienced non-referral and colicky epigastric pain, which gradually declined. About 8 days before admission, our patient became febrile and developed fatigue and myalgia. She complained of nausea, vomiting, anosmia, itching, and skin rashes, which started 3 days before admission. Before the onset of these issues, the patient had been healthy, took no medications, and had no co-morbidities or previous adverse drug reactions. Physical examination revealed a temperature of 38.5 ℃, respiratory rate of 15, pulse rate of 89, O2 saturation of 94%, and blood pressure of 130/90 mm Hg. Moreover, she had icteric sclera. Several painful red nodular lesions were seen on her upper and lower limbs (Figure 1), but no lesions appeared on the palmar and plantar surfaces of her limbs or the patient’s head and neck. As COVID-19 was suspected, a nasopharyngeal swab PCR and multi-slice chest CT scan were requested. Chest CT scan showed bilateral multifocal peripheral ground-glass opacity. Her first laboratory data indicated a white blood cell count (WBC) of 8400× 109/L, lymphocyte = 16%, hemoglobin (Hb) = 11.4 gram/deciliter, platelets count (plt) = 366000, erythrocyte sedimentation rate (ESR) = 36, C-reactive protein (CRP) = 3.3, aspartate aminotransferase (AST) = 78 U/L, alanine transaminase (ALT) = 244 U/Alkaline phosphatase (ALKP) = 966, total bilirubin (total bili) = 11.3, direct bilirubin (direct bili) = 9.8, international normalized ratio (INR) = 1.09, partial thromboplastin time (PTT) = 41, D-dimer = 0.7, creatine (Cr) = 1.1, sodium (Na) = 142, potassium (k) = 4.2, and ferritin = 483. Based on the CT scan and clinical findings, hydroxychloroquine, naltrexone, pantoprazole tablet, cholestyramine, and ceftriaxone injections were prescribed. 

Due to changes in her liver enzymes, a GI consult was requested, after which additional laboratory tests were run, and prednisolone was recommended. On the second day, the patient’s nasopharyngeal swab PCR for COVID-19 was reported positive. Prednisolone was initiated at a dosage of 30 milligrams daily. Abdominopelvic sonography was performed and showed normal size and parenchymal echogenicity for the liver and a CBD size of 6 mm. Neither intrahepatic nor extrahepatic duct dilation was seen. Gallbladder thickness was normal; calculus and sludge were not observed. Normal spleen size and parenchymal echogenicity were reported, and no abnormality in the pancreas, urinary system, or kidneys was seen. On the third day of admission, additional laboratory data showed WBC = 10700, Hb = 11, plt = 247000, ESR = 23, CRP = 3, TSH = 5.6, FT3 = 3.4, FT4 = 1.0, C3 = 105, C4 = 11, HBS Ag = neg, HCV Ab = neg, HAV Ab = neg, HEVAb = neg, anti-rubella IgG = NEG, ANA = 0.4, ANCA = 2.8, AMA = 1.0, AST = 68, ALT = 153, and Alkp = 1353. 

**Figure 2:**
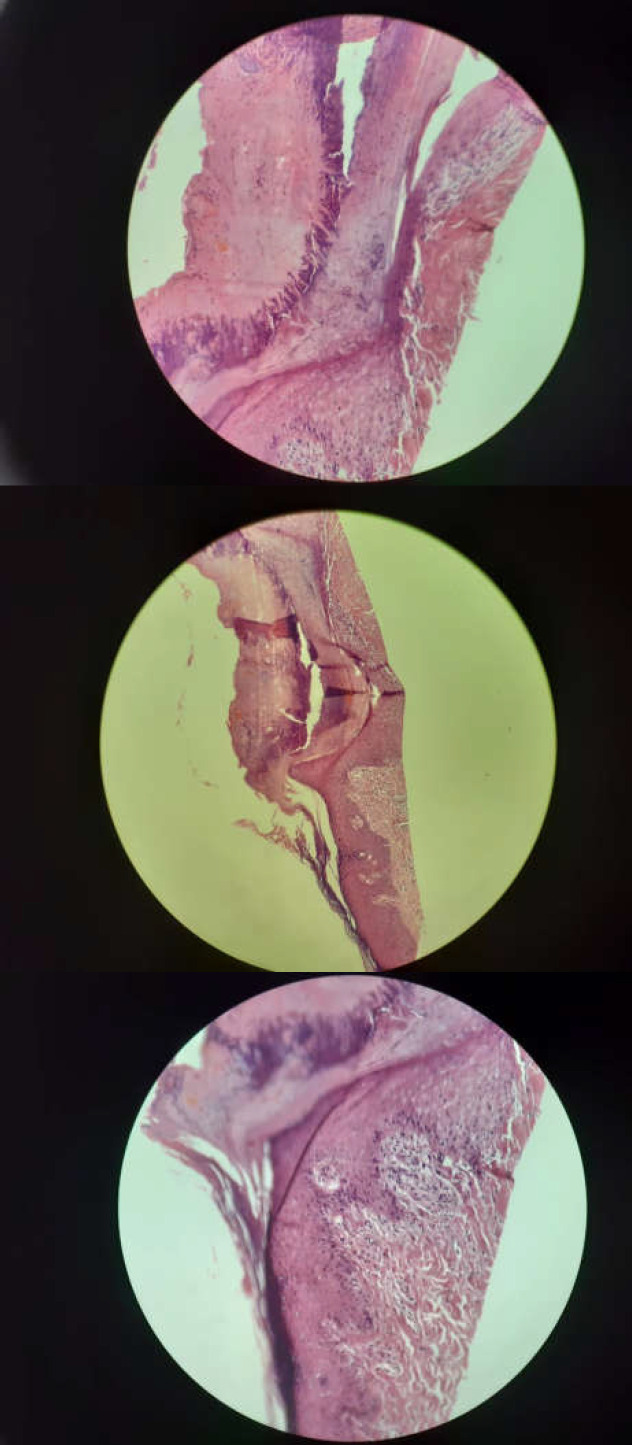
The skin biopsy report showed eosinophil-rich vasculopathy dermal inflammation with focal interface reaction pattern

Additionally, the patient reported increased itching, even though rifampin, hydroxyzine, cholestyramine, and naltrexone had previously been started. Based on the additional laboratory reports, an MRCP and liver biopsy was requested for our patient and the dosages of prednisolone and rifampin increased to 40 mg and 300 mg daily, respectively. 

On the fourth day of admission, the patient was still suffering from pruritus. Because the standard treatment for COVID-19 had failed, the patient was a candidate for plasmapheresis with plasma obtained from previously treated COVID-19 patients.

A central venous catheter was placed and plasmapheresis was initiated and performed three times during the patient’s hospitalization.

On day 12 of admission, the liver pathology report showed severe cholestasis. Four portal spaces were observed with moderated fibrosis and moderate inflammation of chronic inflammatory cells. Lymphocytes and fibroblasts were also presented in the portal spaces. Skin biopsies were done on multiple skin lesions. A new chest CT scan was also performed, and significant recovery in the pulmonary lesions was found.

On day 16 of admission, after 3 plasmapheresis treatments, one unit of washed packed cells was injected into the patient.

On day 17, the skin biopsy report showed eosinophil-rich vasculopathy dermal inflammation with focal interface reaction pattern (Figure 2). The patient was discharged 17 days after admission. After discharge, the patient was followed for about 4 weeks. The corticosteroid was slowly tapered and our patient was symptom-free during the follow-up course.

## Discussion

The pulmonary system is commonly the first site of COVID-19 infection. The results of a meta-analysis showed that fever, cough, and fatigue are the most frequent clinical manifestation in COVID-19 patients. However, other presentations and organ involvement have been reported as well (10). A typical manifestation of COVID-19 was reported in many studies as a first presentation. Therefore, recognizing atypical manifestations of COVID-19 is vital in controlling the outbreak of disease as well as the treatment of COVID-19 patients. 

In our presented case, epigastric pain was the first atypical manifestation. Hormati et al. previously reported that epigastric pain can be one of the gastrointestinal symptoms in COVID-19 patients. Therefore, physicians should be aware of this unusual manifestation (6). Fever and myalgia were the first typical manifestations of COVID-19 in our patient, which started 8 days before admission. Our patient had experienced skin rashes for 3 days prior to admission, and physical examination revealed several painful black nodular lesions on her upper and lower limbs. Several studies have reported the dermatological manifestation of COVID-19 (11). Recalcati et al. found that 20.4% of confirmed COVID-19 patients have a dermatological manifestation and that erythematous rashes were the most common presentation among such skin lesions, which our patient displayed (11). Moreover, similar to the results of Castelnovo et al., a skin biopsy of our patient revealed eosinophil-rich vasculopathy and skin inflammation (12). In their study, COVID-19 skin symptoms manifested as autoimmune disorder-related cutaneous involvement. Therefore, they concluded that the occlusion of small blood vessels plays a crucial role in the cutaneous involvement process in COVID-19 patients (12). Hence, treatment with corticosteroids was initiated according to the previous recommendation of Zhang et al. Treatment with glucocorticoids OM may be useful as immunomodulatory agents in severe COVID-19 complications (13). 

In the case presented herein, liver enzymes including AST, ALT, ALKPH, gamma-GT, and direct bilirubin were significantly increased. Liver cholestasis was suspected; thus, liver biopsy and MRCP were performed. MRCP was reported to be normal, but the liver biopsy showed severe cholestasis. Intrahepatic cholestasis is defined as a condition in which bile flow is decreased (14). Drug-induced and sepsis are common causes of cholestasis and liver injury in COVID-19 patients (15). In drug-induced hepatitis, pruritus is the most common and marked symptom (14). In this type of cholestasis, after stopping drug usage, recovery happens rapidly (14). Moreover, the major histological finding in drug-induced cholestasis is the accumulation of bile inside the hepatocyte and canaliculi, especially in the centrilobular region (14). The present case had no previous history of medication usage; histological findings were different; and liver enzyme changes and pruritus were present at admission time. Accordingly, drug-induced cholestasis may not have been the cause of cholestasis in this patient. In sepsis conditions, the bile acid transportability and bilirubin transport to the canaliculi in the liver are altered following a systemic inflammation response (15). The latent association between some viral infections, including CMV, EBV, and measles (rubella), and intrahepatic cholestasis has been well known (16). Therefore, the ability of COVID-19 to create intrahepatic cholestasis is not surprising. However, previous studies have reported only transient liver enzyme elevation (17). Plasmapheresis treatment was started for our case, which is a useful treatment in COVID-19 as well as cholestasis patients (18, 19).

Herein, we described a COVID-19 patient who had Vasculopathy-related skin lesions, and intrahepatic cholestasis without a history of drug usage. As a result, this case reminded us that in the COVID-19 pandemic, all atypical presentations should be considered as COVID-19 and evaluated. Moreover, liver and cutaneous manifestations of COVID-19 should be noted at the time of diagnosis. However, more investigation is required to clarify all COVID-19 manifestations.

## Conflict of interests

The authors declare that they have no conflict of interest.
